# From pursuing quantity to pursuing quality: exploring the coupling coordination and influencing factors of high-quality tourism development and ecosystem service value in the Pearl River Delta urban agglomeration of China

**DOI:** 10.3389/fpubh.2025.1705088

**Published:** 2025-12-09

**Authors:** Hao Yao, Zeduo Zou, Yiting Zhu, Chunshan Zhou

**Affiliations:** 1 College of Tourism, Xinjiang University, Urumqi, China; 2 School of Geography and Planning, Sun Yat-Sen University, Guangzhou, China

**Keywords:** high-quality tourism development, ecosystem services value, coupling coordination, PRD urban agglomeration, sustainable development theory

## Abstract

**Introduction:**

The coordinated development of tourism and the ecological environment has long been a critical issue for achieving human health and well-being and The United Nations Sustainable Development Goals (SDGs). The tourism industry is gradually shifting its focus from “quantity-driven” to “quality-driven” growth, a necessary transition for its high-quality development. However, few studies have explored the coordinated evolution between the high-quality tourism development and the environment.

**Methods:**

This study introduces the measurement of ecosystem services (ES) to quantitatively assess the spatial–temporal evolution of the coupling coordination degree between high-quality tourism development and ecosystem service value (ESV) in the Pearl River Delta (PRD) urban agglomeration from 2011 to 2022. Furthermore, a panel data model is employed to identify its influencing factors.

**Results and discussion:**

The findings are as follows: (1) the comprehensive index of high-quality tourism development exhibited a trend of an initial rise followed by a decline, displaying a spatial distribution pattern of “high in the central region, low in the eastern and western regions.” (2) The overall ESV showed a fluctuating downward trend, with a spatial pattern of “low in the east, high in the west; high in the north, low in the south.” (3) The coupling coordination effect first increased and then decreased, having long been in a run-in phase, presenting a spatial distribution of “high in the north, low in the south; high in the west, low in the east.” (4) The coordinated development of the two systems is influenced by economic development, governmental environmental regulations, urban expansion, and industrial structure. The results of this research provide significant theoretical and practical implications for the coordinated development of tourism system and ecosystem services.

## 1 Introduction

The harmonious coexistence between humans and nature is a critical issue of concern across society. As an industry with a strong dependence on the ecological environment, tourism is inextricably linked to it. On one hand, tourism activities can cause certain disturbances to the destination’s ecosystem (1). On the other hand, the economic benefits generated from tourism development provide financial support for ecological maintenance and restoration (2). Concurrently, a high-quality ecological environment, serving as a core attraction, can effectively enhance tourist satisfaction (3), thereby strengthening tourism competitiveness and promoting the industry’s sustainable development.

With the environmental and social turn in tourism studies, the relationship between tourism and the ecological environment has become a central issue in the field (4). Relevant research has primarily focused on two levels: individual and local. From the perspective of the individual, studies have concentrated on the practices of stakeholders (e.g., tourists, residents, and tourism practitioners) in managing the relationship with environmental protection during tourism activities. This includes the specific manifestations, driving factors, and generation mechanisms of individual pro-environmental behavior, as well as the game between limited tourism development and environmental conservation among stakeholders (5–7). From the local perspective, scholars have been investigating concepts such as the environmental carrying capacity and sustainable development of tourist destinations since the 1980s. As the negative environmental impacts of tourism have deepened, scholars have begun to reflect on this extensive, resource-intensive model of tourism economic development, shifting their focus to the coordinated relationship between tourism development and the ecological environment (8).

The study on the coordination between tourism development and the ecological environment reveals several characteristics. First, regarding research methodology, the coupling coordination model is the most widely applied. For instance, Tang explored the (CCD) between tourism industry development and the environment in Heilongjiang Province in China, noting that the degree of coordination was increasing annually, influenced by economic benefits and ecological quality (9). Second, research scales have spanned micro, meso, and macro levels, exploring the relationship at various scales including tourist destinations, urban agglomerations, and provincial regions, with study areas such as Wuling Mountain, Chongqing City, the Yellow River Basin, and Heilongjiang Province (8–12). Third, existing studies often rely on the comprehensive index method to measure the indirect relationship between the two systems, lacking direct and scientific measurement approaches (13, 14). Fourth, much of the research is descriptive, focusing on the state of the coordinated relationship, with less attention paid to the factors that influence it.

Ecosystem services (ES) are defined as the direct or indirect benefits that humans obtain from ecosystems (15), and a mature research framework has since been developed. Firstly, compared to the comprehensive index method, ESV can more directly and objectively reflect the quality of the ecological environment, addressing the issue of subjective judgment by researchers in environmental assessment (16). Secondly, the ecological environment is essentially a public resource, characterized by non-excludability and non-rivalry in consumption, and is thus often accompanied by externalities (17). However, existing research has predominantly focused on the direct effects of the environment on humans, while paying less attention to indirect effects such as the hidden costs of environmental degradation or the added benefits of ecological improvement (18). The advantage of the ES framework is its ability to not only capture these direct effects but also to incorporate and quantify these often-overlooked indirect externalities. Lastly, traditional ecological indicators are difficult to compare directly with tourism development data due to their disparate units. In contrast, ES utilizes monetization to convert ecological costs and benefits into a unified value scale, enabling cross-indicator and cross-regional comparability and integration (19).

The coordinated relationship between the tourism industry and ecosystem service value (ESV) provides an important bridge for understanding the relationship between human activities and nature. The CCD plays a vital role in exploring this relationship (9). Current research on this topic has been conducted at various scales, from tourist destinations to provincial levels, and has characterized tourism development using diverse indicators such as tourism development, tourism economy, tourism economic resilience, and tourism urbanization (8, 10, 12, 20). For example, Huang et al. (8) investigated the coupling coordination between China’s tourism economy and ESV, finding that the two systems were in a long-term, and strengthening, phase of high-level adjustment. However, as societal expectations for the tourism industry shift from quantity to quality (21), traditional metrics like the tourism economy are no longer sufficient to meet the demands of China’s pursuit of high-quality development. High-quality tourism development, as a comprehensive and sustainable assessment approach, integrates concepts of economic growth, social equity and sharing, environmental sustainability, and cultural preservation. It advocates for the sustainability of tourism development as a response to the limitations of focusing solely on economic growth (22, 23). This concept aligns seamlessly with the principles of sustainable development theory. Therefore, understanding the coordinated relationship between high-quality tourism development and ESV can effectively assess the current state of synergy between tourism and the environment, thereby advancing the nation’s high-quality development in the new era.

Based on this, the present study introduces ESV into the research on the coordinated development of high-quality tourism and the ecological environment. This allows for a more scientific and objective measurement of the functional services that ecosystems provide for social development, advancing empirical research in this area. The PRD urban agglomeration, leveraging its locational advantages and economic strength, has achieved rapid tourism growth. However, rapid urbanization has led to a significant reduction in cultivated land and water bodies, alongside a swift expansion of construction land, thereby impacting the ecological environment and posing great challenges to its sustainable development (24). As one of the most representative study areas, this research selects the PRD urban agglomeration as its case. It employs a CCD model to measure the overall trends, the evolutionary characteristics of different cities, and the changes in coordination between high-quality tourism development and ESV from 2011 to 2022. Furthermore, a panel data model is used to analyze the factors influencing these changes, with the aim of providing a scientific basis for the sustainable development pathway of tourism in the PRD urban agglomeration.

## 2 The coupling mechanism of high-quality tourism development and ESV based on the theory of sustainable development

Ecosystem services (ES) create an essential link between human society and the natural environment, playing a crucial role in determining the harmonious coexistence of tourism activities and nature (15). The benefits that tourists derive from nature are a direct manifestation of how ES enhance human well-being, positioning the tourism industry as a key “beneficiary” (8). Conversely, the tourism industry reshapes the generation of ES, meaning that tourism both depends on and continuously redefines the ecosystem. When tourism expansion is excessively rapid, the trajectory of ES can become uncertain. This bidirectional interaction makes the relationship between the two systems considerably more complex.

**Figure 1:**
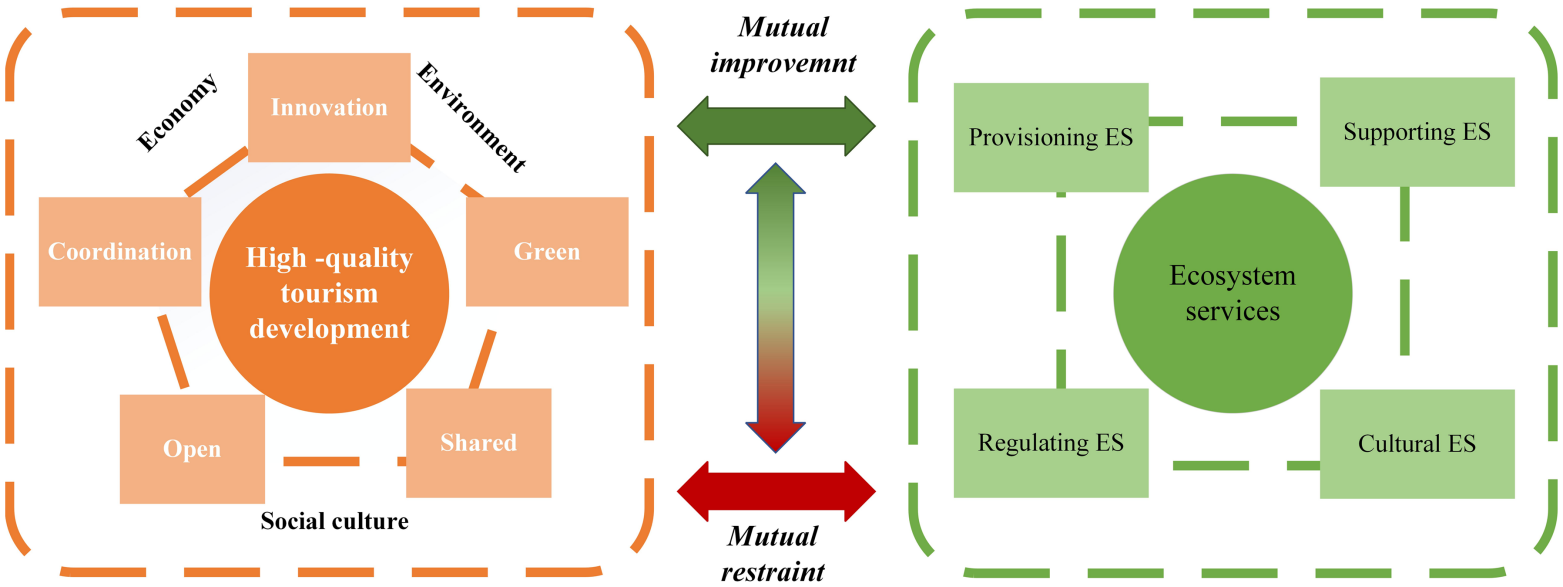
Coupling mechanism diagram of high-quality tourism development and ESV.

A clear connection exists between high-quality tourism development and ecosystem ESV. China’s national development philosophies, such as “high-quality tourism development” and the principle that “lucid waters and lush mountains are invaluable assets,” have forged a closer bond between tourism and the natural environment. From a systems theory perspective, high-quality tourism development and ES can be viewed as two interacting subsystems that mutually influence each other and exhibit dynamic changes across spatio-temporal scales. High-quality tourism development is an advanced expression of sustainable development; both concepts fundamentally pursue the comprehensive development of human beings and adhere to the three bottom lines of economic, environmental, and socio-cultural sustainability (25). Therefore, drawing upon the framework of sustainable development theory and integrating China’s five concepts for development (innovation, coordination, green, openness, and sharing), this study proposes the coupling mechanism between high-quality tourism development and ESV (Figure 1).

From an economic sustainability perspective, high-quality tourism development emphasizes innovative, open, and coordinated regional tourism development. Innovation makes the implicit values of ES explicit through advanced technologies and management models. Openness amplifies the “spillover radius” of ESV via the tourism market. Coordination, through integrated regional tourism strategies, secures or enhances ESV to achieve long-term viability. From socio-cultural and environmental sustainability perspectives, the concept of sharing promotes the shared prosperity of tourism products and services, while green development mandates environmental sustainability. Together, they drive the preservation and enhancement of ES through the effective conservation of tourism resources, leading to relative stability and continuous value appreciation (26).

Conversely, ES contribute to high-quality tourism development by providing stable, explicit, and long-term benefits. Provisioning services offer material support for high-quality tourism development, such as food, fresh water, and energy for tourists. Regulating, supporting, and cultural services provide the foundational ecological spaces and tourism resources. For instance, a healthy atmospheric environment and rich biodiversity enhance tourists’ travel intentions and subsequently boost the destination’s reputation and appeal (27).

Therefore, the mutual coordination and promotion among the constituent elements of the high-quality tourism development and ES subsystems are essential for achieving a more sustainable form of tourism.

## 3 Research design

### 3.1 Research area

**Figure 2:**
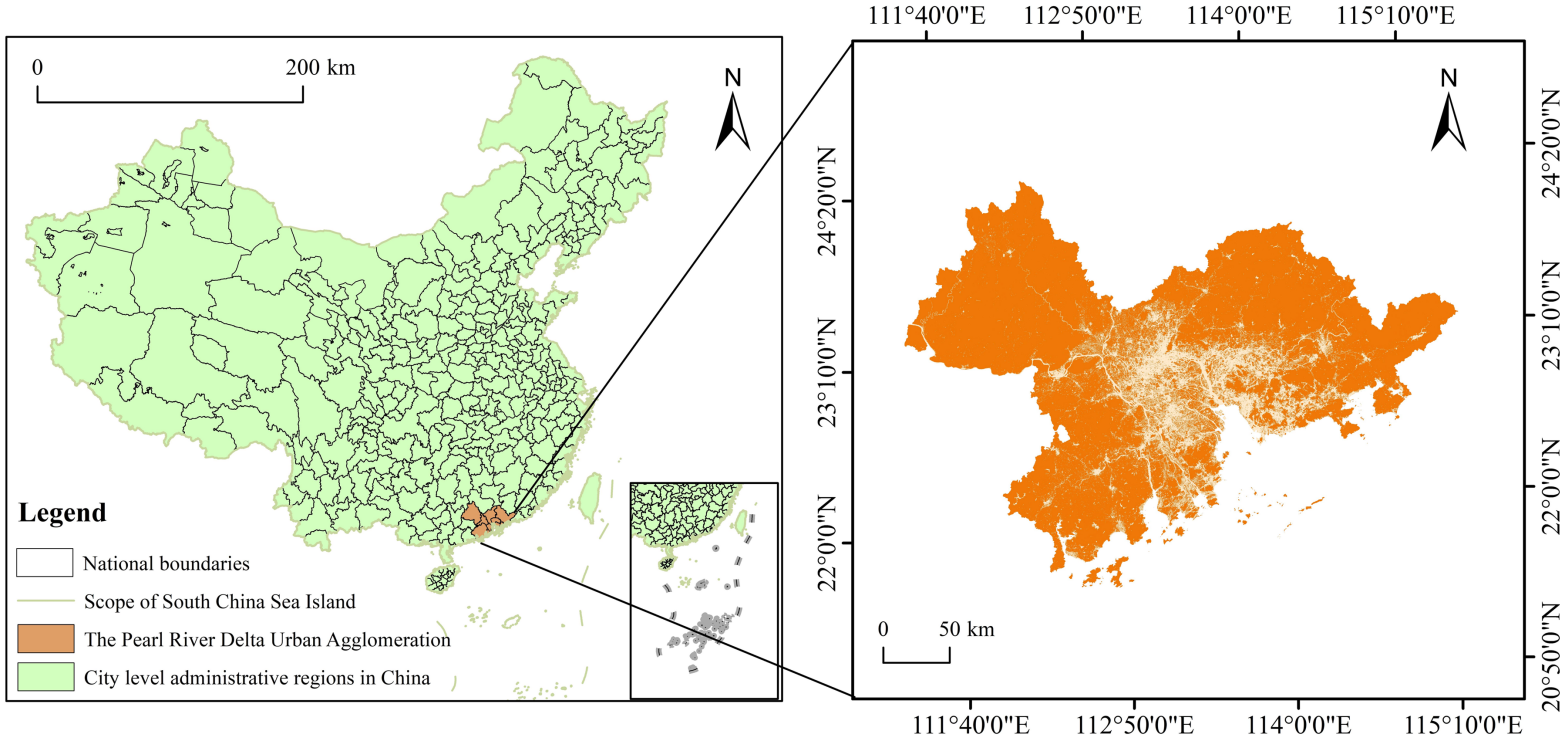
Location of PRD urban agglomeration. This map is based on the standard map with the review number GS (2019)1822 downloaded from the Standard Map Service Website of the Ministry of Natural Resources in China. The boundaries of the base map have not been modified.

The PRD urban agglomeration is located in the south-central part of Guangdong Province, China (111°20′–115°25′E, 21°27′–23°57’ N) (Figure 2). It is composed of nine cities: Guangzhou, Shenzhen, Zhuhai, Zhaoqing, Foshan, Huizhou, Jiangmen, Dongguan, and Zhongshan. The PRD urban agglomeration has a total area of 5.47 million hectares, of which 30% consists of mountains, hills, and islands, and it features a subtropical monsoon climate. The region’s ecosystem structure is characterized by significant diversity, with forests and water bodies being its primary components.

The rapid development of tourism in the PRD was catalyzed by China’s Reform and Opening-Up policy. After the 1990s, following a structural economic adjustment, the region’s focus shifted from traditional sightseeing to high-tech industries, cultural and creative industries, and modern service sectors. This transition spurred the growth of business tourism and the MICE (Meetings, Incentives, Conferences, and Exhibitions) industry. The promulgation of the Tourism Integration Plan for the PRD urban agglomeration (2014–2020) in 2014 further advanced this development by promoting tourism cooperation among the nine cities with the goal of establishing a world-class tourist destination. Today, the PRD urban agglomeration stands as one of China’s most developed regions in terms of tourism economy. Its tourism revenue grew from 374.613 billion yuan in 2015 to 1,008.668 billion yuan in 2019. Subsequently, the COVID-19 pandemic exerted a negative impact on the global tourism industry, and the PRD urban agglomeration was no exception. Throughout this development process, the region has also experienced progressive urban expansion and rising urbanization levels, leading to an increase in land use for tourism and other construction, thereby facing increasingly severe environmental challenges (24).

### 3.2 Data sources

Data regarding the high-quality tourism development in this study were mainly sourced from the *China City Statistical Yearbook*, the *Guangdong Statistical Yearbook*, and the annual statistical bulletins of the nine prefecture-level cities, such as domestic tourism revenue, etc. Additionally, the number of tourism patent applications was obtained from the Dudu Patent Search Network (https://www.iprdb.com). The number of employees in the tourism sector was calculated as the sum of personnel in the accommodation and catering industry and the culture, sports, and entertainment industry. To ensure the authenticity and validity of the indicators, and differing from previous research, this study uses the number of overnight inbound tourists to represent the total inbound tourist arrivals.

For the ESV data, this study utilized land use data from 2011 to 2022 from the China Land Cover Dataset (CLCD, https://zenodo.org/records/8176941), which has an overall accuracy of 80%. Preprocessing of the annual remote sensing images was conducted using ENVI 5.1 software. In accordance with China’s Land Use Status Classification Standard (GB/T21010-2017), visual interpretation was performed, supplemented by field surveys and the use of Google Earth. The maximum likelihood classification method was employed to classify the study area, which was then resampled into seven categories: cropland, forests, grassland, water bodies, wetlands, construction land, and unused land.

Furthermore, the relationship between high-quality tourism development and ESV is intrinsically linked to the foundational advantages of each locality. Drawing upon existing research relevant to the specific context of the PRD urban agglomeration (24, 28–30), this study selected four indicators to measure the factors influencing the CCD between high-quality tourism development and ESV: economic development, industrial structure, government environmental regulation, and urban expansion. Among these: GDP per capita to represent the economic development (9, 31); the proportion of the added value from the tertiary industry to represent the industrial structure (32); and an index for the intensity of municipal government environmental regulation, constructed based on the frequency of terms related to “environmental protection” in annual government work reports (33). Urban expansion was represented by the average intensity of nighttime lights (34, 35). Data for economic development and industrial structure were obtained from the *Guangdong Statistical Yearbook*, while the average nighttime light data were sourced from the Harvard Dataverse platform. Due to data acquisition limitations, missing data points were filled using the linear interpolation method, while data that could not be directly obtained were derived through the conversion of relevant raw data.

### 3.3 Research methods

#### 3.3.1 Method for evaluation of the high-quality tourism development

High-quality tourism development is a rich and multifaceted concept. Drawing upon the analytical framework of Zhang and Yang (25) and grounded in sustainable development theory alongside China’s five concepts for development (innovation, coordination, green development, openness, and sharing), this study constructs an indicator system for high-quality tourism development. Additionally, by referencing relevant literature, we established an evaluation framework for high-quality tourism development comprising three dimensions, five sub-dimensions, and 21 indicators (8, 9, 22, 32, 36), as detailed in Table 1. The calculation steps are as follows:

**Table 1:** Construction of the index system for high-quality tourism development based on the sustainable development theory.

Dimension 1	Dimension 2	Indicator	Indicator explanation	Indicator attribute	Source
Economical level	Tourism Innovation Development	Tourism investment and research expenditure	R&D expenditure × (total tourism revenue / GDP) (in ten thousand yuan)	+	(36)
		Tourism innovation products	The number of tourism patents held by 10,000 people (in pieces)	+	(36)
	Tourism openness level	The number of inbound arrivals	The number of overnight inbound visitors	+	(22)
		Inbound consumption	Inbound tourism revenue	+	(8)
	Tourism area coordination	Contribution of tourism to the economy	Domestic tourism revenue	+	(9)
			The proportion of tourism revenue in the GDP	+	(9)
		Contribution of tourism to employment	The proportion of tourism industry workers in the total number of employees	+	(37)
		The contribution of tourism industry linkage	The proportion of tourism revenue in the output value of the tertiary industry	+	(8)
Social cultural level	Tourism products and services sharing	Density of high-quality tourism resources	Number of 4A and 5A-level scenic spots (places)	+	(37)
		High-quality accommodation supply for tourism	Number of four-star and five-star hotels (places)	+	(37)
		Tourism public transportation services	The number of taxis in operation per capita at the end of the year (in units of vehicles)	+	(36)
			The number of actual public transport vehicles in operation per capita at the end of the year	+	(36)
		Tourism Public Health Services	The number of public toilets (facilities) per 10,000 people	+	(32)
		Tourism cultural facility services	Number of museums	+	(22)
			Number of public libraries	+	(22)
			Number of art performance groups and institutions	+	(22)
Environmental level	Tourism green development	Park green space area	Per capita park green space area (m^2^)	+	(32)
		Greening of the built-up area	Green coverage rate of built-up area (%)	+	(32)
		Level of air pollution	Annual average PM2.5 concentration	–	(36)
		Garbage harmless treatment	Garbage harmless treatment	+	(32)
		Wastewater treatment	Wastewater treatment rate	+	(36)

The Entropy Weight Method (EWM) is an objective weighting model widely utilized in multi-criteria comprehensive evaluation. The value of the EWM lies in its ability to optimize two critical aspects of the assessment process simultaneously. On one hand, it prevents the interference of subjective judgment in weight allocation. On the other hand, it resolves potential issues of high collinearity or information redundancy that can arise when numerous evaluation indicators are involved, thereby ensuring the robustness of the results. Therefore, this study selects the EWM, in conjunction with the established indicator system, to measure the high-quality tourism development and ESV in the PRD from 2011 to 2022. The specific steps are as follows:

1. Standardization of indicator data: the raw data for the constructed indicators are standardized using the Equations (1,2):

$$y_{\mathrm{ij}}=\frac{x_{\mathrm{ij}}-x_{j\min}}{x_{j\max}-x_{j\min}}\;(\text{positive indexes})$$ (1)

$$y_{\mathrm{ij}}=\frac{x_{j\max}-x_{\mathrm{ij}}}{x_{j\max}-x_{j\min}}\;(\text{negative indexes})$$ (2)

Where *x_ij_* is the original data matrix of the index; *y_ij_* is the standardized data matrix; *x_jmax_* and *x_jmin_* are the maximum and minimum values of the *j*th indicator, respectively.

1. Calculation of indicator entropy: the entropy for each indicator is calculated with the Equations (3,4):

$$Y_{\mathrm{ij}}=\frac{y_{\mathrm{ij}}}{\sum_{i=1}^{m}y_{\mathrm{ij}}}$$ (3)

$$e_{j}=-\frac{1}{\ln m}\sum_{i=1}^{m}Y_{\mathrm{ij}}\ln Y_{\mathrm{ij}}$$ (4)

Where *ej* is the entropy value of the *j* indicator, and *Yij* represents the proportion of the value. For the *i* year under the *j* indicator relative to the sum of all values for that indicator.

1. Calculation of indicator weights: the weight value for each indicator is computed using the Equations (5–6):

$$d_{j}=1-e_{j}$$ (5)

$$W_{j}=\frac{d_{j}}{\sum_{j=1}^{n}d_{j}}$$ (6)

Where *d_j_* represents information utility value, and *W_j_* is the indicator weights.

1. Calculation of the comprehensive evaluation index: the index for each year is calculated as Equation (7):

$$S_{i}=\sum W_{j}y_{\mathrm{ij}}$$ (7)

Where *S_i_* represents comprehensive evaluation scores, and *W_j_* is the indicator weights.

#### 3.3.2 ESV assessment method

The quantitative assessment of ESV originated from the seminal work of Costanza et al. (15). However, the direct application of this global model to China is inadequate due to the regional specificity of its ecosystems and functional services. To address this limitation, Xie et al. developed a localized modification of the original theory (37), proposing a system of equivalent value factors per unit area suited to China. Following the methodology, this study defines the base equivalent factor of ESV as one-seventh of the average economic value of grain production per unit area in Guangdong Province. The Equation (8) is as follows:


$$E_{a}=\frac{1}{7}\times \frac{\mathrm{PQ}}{S}$$ (8)

Where *Ea* is the economic value of grain production per unit area of cropland (yuan/ha); *P* is the average grain purchase price (yuan/kg); *Q* is the total grain yield (kg); and *S* is the total sown area of grain crops (ha). Based on national grain purchase price data, the average value of *P* was determined to be 3.05 yuan/kg. This yields an economic value per unit area of cropland of 2,418.58 yuan/ha. This value was then adjusted using a correction coefficient, *λ*, calculated as the ratio of the annual average grain yield per unit area in the study area to the national average grain yield per unit area (λ = 0.968). Finally, corrected economic value for a unit of cropland in the PRD was thus calculated to be 2,341.52 yuan/ha.

From this base value, the ESV coefficient *VC* for each ecosystem service can be calculated according to the table of equivalent factors. While previous studies commonly assigned a zero value to the ESV of construction land, recent research has found that it provides certain ecological functions, such as cultural and recreational services. Based on the relevant research (24), the service values per unit area for the ecosystems in the PRD are summarized in Table 2. The calculation of the value coefficient is as shown in Equation (9):


$$VC_{\mathrm{ij}}=e_{\mathrm{ij}}\times E_{a}(i,j=1,2\cdots ,n)$$ (9)

**Table 2:** The ESV per unit area in the PRD.

Dimension	Indicator	Cropland	Forests	Grassland	Water bodies	Construction land	Wetlands	Unused land
Provision services	Food products	3184.46	679.04	889.77	1873.21	0	1194.17	0
	Material products	210.73	1545.40	1311.25	538.54	0	1170.76	0
Regulating services	Water supply	−6158.19	796.11	725.87	19411.20	−17584.8	6064.53	0
	Gas regulation	2599.08	5081.09	4612.79	1802.97	−5666.48	4448.88	46.83
	Climate regulation	1334.66	15219.88	12199.32	5362.08	0	8429.47	0
	Environmental purification	398.05	4519.13	4027.41	12995.44	−5760.14	8429.47	234.15
	Hydrological regulation	6368.93	11098.80	8944.60	239397.00	0	56735.03	70.24
	Soil conservation	23.41	6205.02	5619.64	2177.61	46.83	5408.91	46.83
Supporting services	Maintenance of nutrient cycling	444.88	468.30	421.47	163.90	0	421.47	0
	Maintenance of biodiversity	491.71	5643.06	5104.51	5970.87	796.11	18427.76	46.83
Cultural services	Aesthetic landscape	210.73	2482.01	2247.85	4425.47	23.41	11075.39	23.41
Total		9108.45	53737.84	46104.48	294118.29	−28145.06	121805.84	468.29

Where *e_ij_* represents the equivalent factor for service category *j* of land use type *i*.

Based on the land use data and the calculated table of value coefficients per unit area, the total ESV for the PRD for each year is calculated using the Equation (10):


$$\mathrm{ESV}=\sum_{i=1}^{n}VC_{i}\times A_{i}$$ (10)

#### 3.3.3 Coupling coordination degree model

The Coupling Coordination Degree model is employed to measure the level of coordinated development between the tourism system and the environmental system (9). In this study, the CCD model is adopted to investigate the synergistic evolutionary relationship between high-quality tourism development and ESV. The specific calculation Equations (11–13) are as follows:


$$C=2\times \sqrt{\frac{f(x)\cdot g(x)}{{\left(f(x)+g(x)\right)}^{2}}}$$ (11)

T=αf(x)+βg(x) (12)

$$D=\sqrt{C\times T}$$ (13)

Where *C* represents the coupling degree, *T* is the comprehensive coordination index, and *D* is the CCD. *α* and *β* are the weighting coefficients. As high-quality tourism development and ESV are considered equally important in this research, the coefficients are set as *α = β =* 0.5.

Furthermore, by referencing the classification standards for CCD from (38) and adapting them to the specific conditions of the PRD region, the types of coupling coordination between high-quality tourism development and ESV are subdivided into three stages and nine types (Table 3).

**Table 3:** Classification criteria for CCD of high-quality tourism development and ESV in the PRD urban agglomeration.

Stage	Type	CCD	Stage	Type	CCD
Disorder	Severe disorder	0.00–0.19	Coordination	Elementary coordinate	0.60–0.69
	Moderate disorder	0.20–0.29		Intermediate coordinate	0.70–0.79
	Mild disorder	0.30–0.39		Good coordinate	0.80–0.89
Run-in stage	Borderline disorder	0.40–0.49		Excellent coordinate	0.90–1.00
	Bare coordinate	0.50–0.59			

#### 3.3.4 Model selection of influencing factors

Drawing on existing research (24, 28–30), this study conducted a multicollinearity test on the panel data for four indicators of the PRD urban agglomeration from 2011 to 2022: economic development, industrial structure, government environmental regulation, and urban expansion (Table 4). The VIF (Variance Inflation Factor) values for all four indicators were less than 10. This result indicates that all four indicators passed the multicollinearity test and can therefore be used in the subsequent panel model analysis.

**Table 4:** Collinearity diagnostics.

Items	VIF	Tolerance
Industrial structure *(IS)*	1.473	0.679
Government environmental regulation *(GER)*	1.104	0.906
Urban expansion *(UR)*	2.214	0.452
Economic development *(ED)*	1.823	0.549

Panel data models offer several advantages, including the ability to incorporate more sample information, facilitate dynamic analysis over time, and effectively mitigate problems of endogeneity. In this study, a comparison was made among the fixed-effects, random-effects, and pooled models. The random-effects model was ultimately selected, given that it utilizes both within-group and between-group variation, allows for the estimation of time-invariant variables, demonstrates greater efficiency with the same sample size, and its results can be directly generalized to the population (39).

Model selection was conducted using the F-test, the Breusch-Pagan Lagrange Multiplier (LM) test, and the Hausman test. The results are presented in Table 5. The *p*-values for both the F-test and the Breusch-Pagan LM test were significant, while the p-value for the Hausman test was not significant. This outcome confirms the appropriateness of the random-effects model for the analysis.

**Table 5:** Summary of test results.

Type of test	Purpose of the test	Value	Test conclusion
F-test	Comparison and Selection between FE Model and POOL Model	*F* (8,95) = 76.839, *p* = 0.000	FE Model
BP-test	Comparison and Selection between RE Model and POOL Model	χ^2^ (1) = 397.269, *p* = 0.000	RE Model
Hausman-test	Comparison and Selection between FE Model and RE Model	χ^2^ (3) = 1.809, *p* = 0.613	RE Model

Based on the above, a random-effects model was constructed to analyze the factors influencing the CCD. The specific Equation (14) of the model is as follows:


$$D_{\mathrm{it}}=\beta_{0}+\beta_{1}ED_{1\mathrm{it}}+\beta_{2}IS_{2\mathrm{it}}+\beta_{3}\mathrm{GE}R_{3\mathrm{it}}+\beta_{4}UE_{4\mathrm{it}}+u_{i}+\varepsilon_{\mathrm{it}}$$ (14)

Where *β* represents the coefficient, *i* indicates each city, *t* represents each year, μ *_i_* denotes the random effect term, and ε *_it_* is the error term.

## 4 Research findings

### 4.1 Spatial and temporal evolution of the high-quality tourism development level in the PRD urban agglomeration

#### 4.1.1 Temporal evolution of the high-quality tourism development level in the PRD urban agglomeration

**Figure 3:**
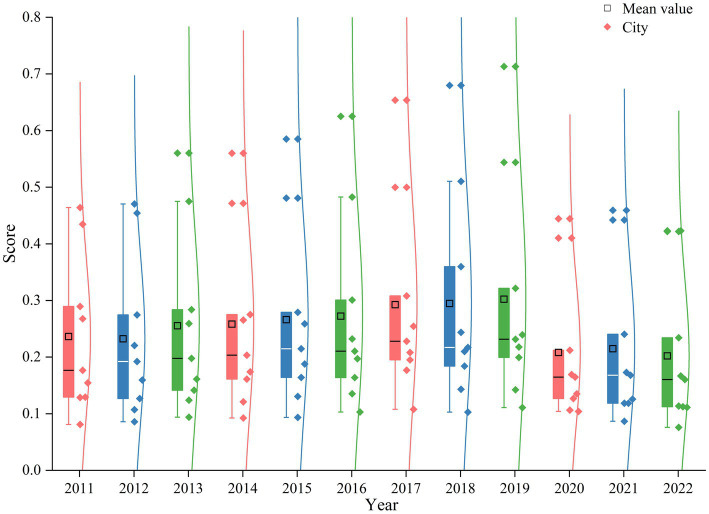
The overall trend of high-quality tourism development in the PRD urban agglomeration from 2011 to 2022.

The comprehensive evaluation index for the high-quality tourism development in the PRD urban agglomeration was measured, with the results depicted in the figure. Figure 3 presents a box plot of the high-quality tourism development index. From an overall perspective, the average level of high-quality tourism development fluctuated between 0.20 and 0.29 during the study period, exhibiting a general trend of a slow increase followed by a decline. Specifically, the index rose gradually from 2011 to 2019. Existing research on the PRD urban agglomeration indicates that the region entered an early-stage advanced economy as early as 2012 (24). This economic development has driven up urbanization levels, resulting in more comprehensive supporting tourism infrastructure compared to other regions. Consequently, the level of high-quality tourism development in the PRD peaked in 2019. Beginning in 2020, due to the impact of pandemic-related lockdowns and controls, the domestic tourism industry suffered a significant shock, leading to a progressive decrease in the level of high-quality tourism development. From the perspective of individual cities, the level of high-quality tourism development across the PRD urban agglomeration is highly uneven. From 2011 to 2019, the internal disparities in the comprehensive evaluation index among the nine cities gradually widened. Conversely, from 2020 to 2022, under the impact of the pandemic, these internal differences in development levels progressively narrowed.

#### 4.1.2 Spatial distribution of high-quality tourism development in the PRD urban agglomeration

**Figure 4:**
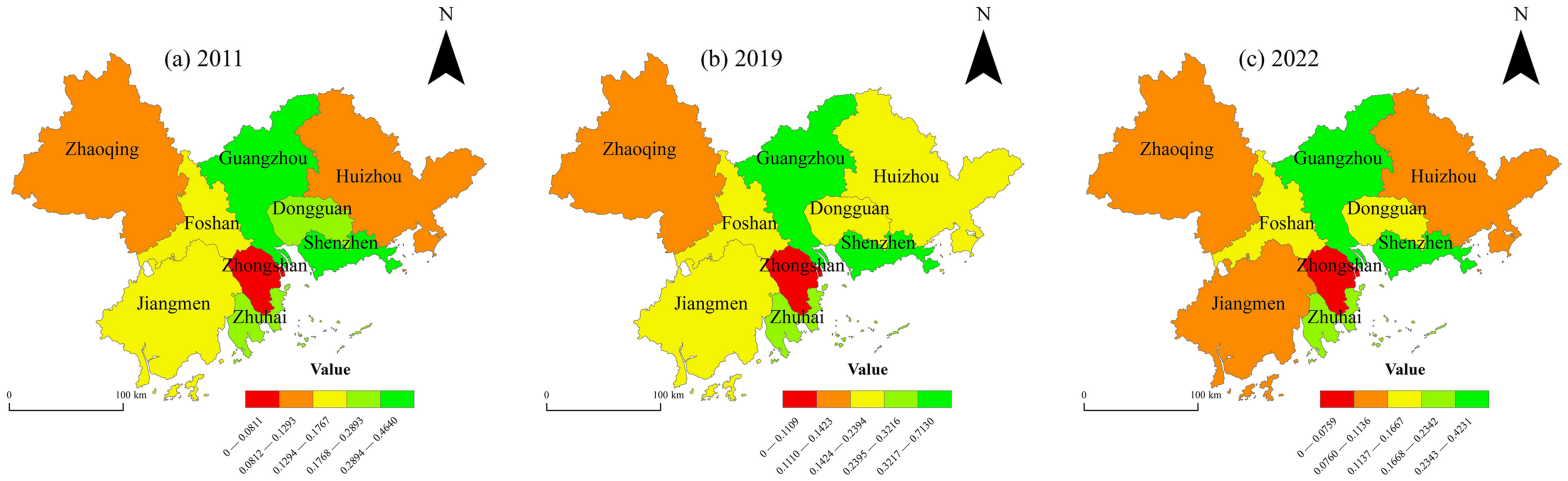
Spatial distribution of high-quality tourism development in the PRD urban agglomeration from 2011 to 2022. This map is based on the standard map with the review number GS (2019)1822 downloaded from the Standard Map Service Website of the Ministry of Natural Resources in China. The boundaries of the base map have not been modified.

Data on high-quality tourism development for the years 2011, 2019, and 2022 were selected to analyze the evolutionary characteristics of the spatial pattern across the nine cities of the PRD urban agglomeration, using ArcGIS 10.8 software. The results are illustrated in Figure 4. Overall, the level of high-quality tourism development in the PRD urban agglomeration experienced a trend of an initial increase followed by a decline. The spatial distribution exhibits a distinct pattern of being “high in the central region and low in the eastern and western regions.” Notably, the levels in Guangzhou, Shenzhen, and Zhuhai are significantly higher than in other areas, forming an evolutionary pattern that diffuses outwards from these three cities as the core. This can likely be attributed to their strong economic foundations, comprehensive tourism infrastructure, and superior quality of tourism services.

In 2011, the level of high-quality tourism development across the PRD urban agglomeration showed considerable spatial differentiation, with Shenzhen and Guangzhou taking the lead while other cities lagged. By 2019, the overall level had risen, and the development index showed a marked improvement, indicating a positive development trajectory for the region’s tourism industry. In 2022, impacted by the COVID-19 pandemic, the level of high-quality tourism development in cities such as Jiangmen and Huizhou declined. In contrast, Guangzhou, Shenzhen, and Zhuhai maintained a certain degree of tourism resilience within the PRD urban agglomeration.

### 4.2 Spatial and temporal evolution of ESV in the PRD urban agglomeration

#### 4.2.1 Temporal evolution of ESV in the PRD urban agglomeration

**Figure 5:**
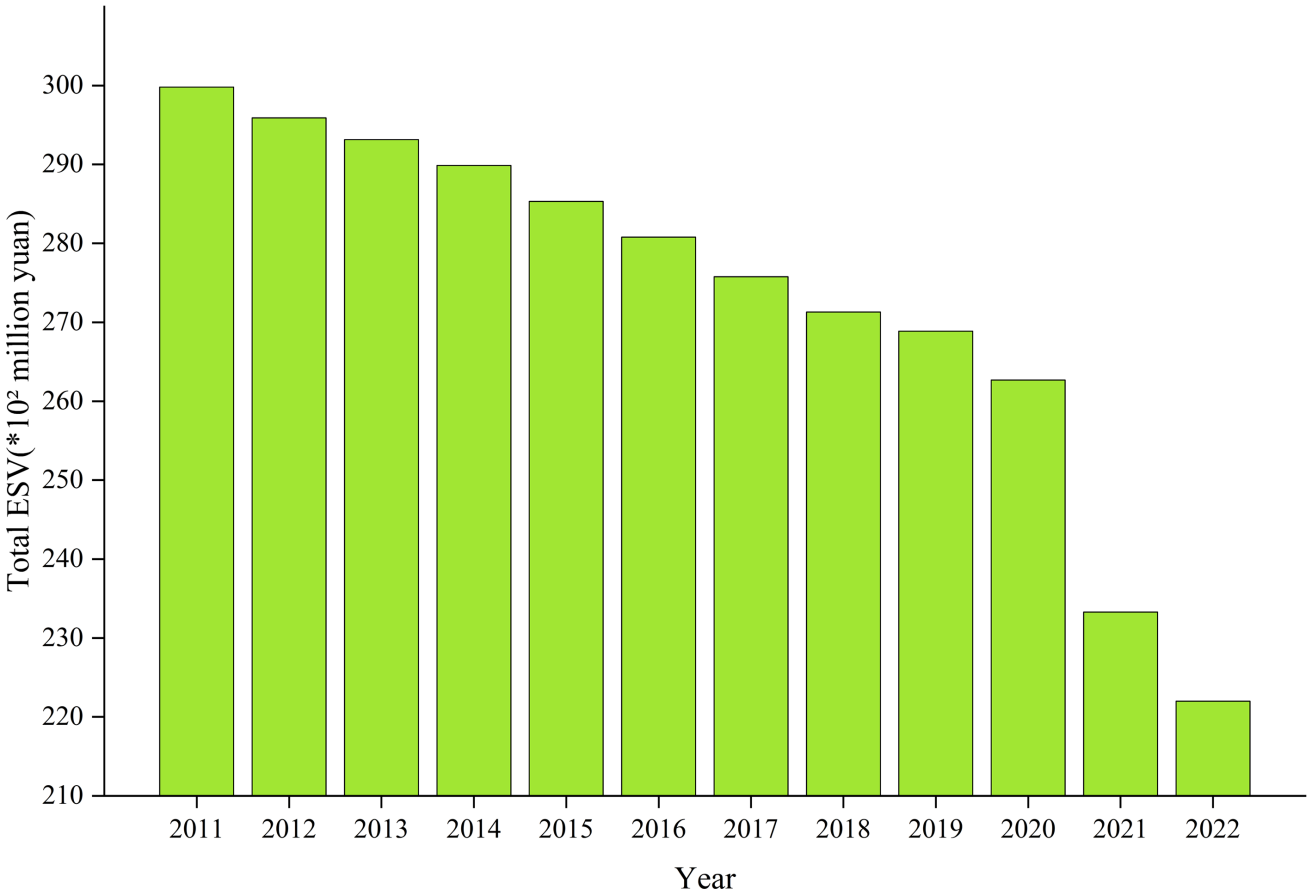
Overall trend of ESV in the PRD urban agglomeration from 2011 to 2022.

ESV exhibits distinct characteristics at different stages of urbanization (24, 40). During the study period, the total ESV in the PRD urban agglomeration showed a declining trend (41). From an urbanization perspective, the PRD urban agglomeration was in the mature stage of post-urbanization during this time. The ESV contributed by construction land and unused land increased overall, whereas the ESV from cropland, forests, grassland, and water bodies generally decreased. It is evident that fluctuations in cropland, forests, water bodies, and construction land influenced the overall trajectory of the total ESV in the PRD urban agglomeration. This may indicate that the encroachment on cropland, forests, and water bodies during urban expansion is the key driver behind the reduction in ecosystem service functions (Figure 5).

#### 4.2.2 Spatial distribution of ESV in the PRD urban agglomeration

**Figure 6:**
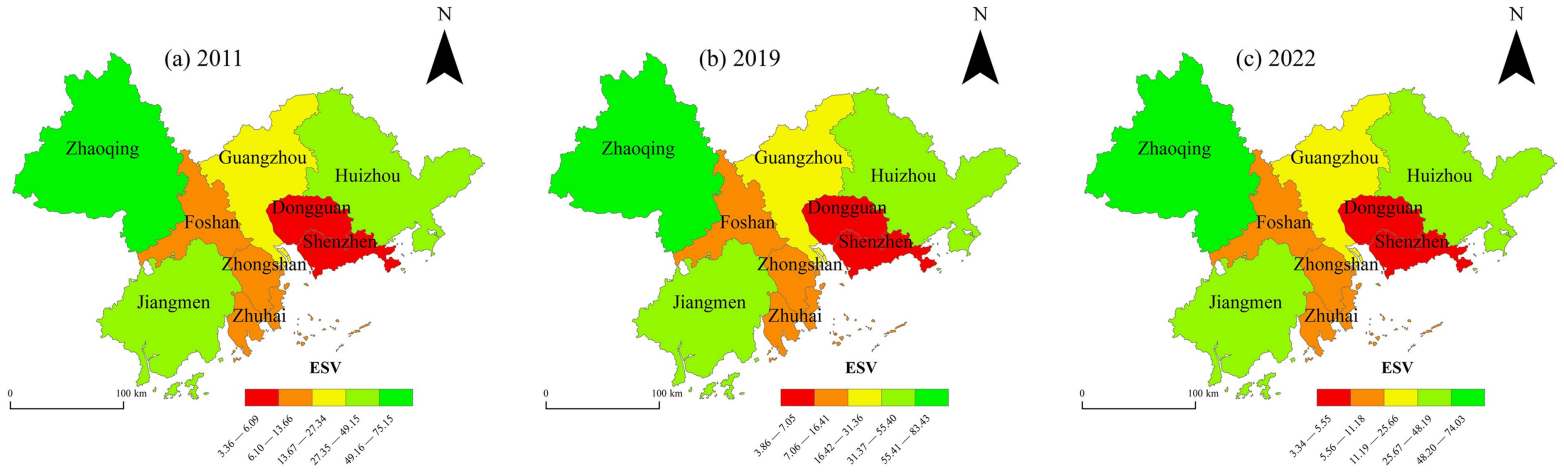
Spatial distribution of ESV in the PRD urban agglomeration from 2011 to 2022. This map is based on the standard map with the review number GS (2019)1822 downloaded from the Standard Map Service Website of the Ministry of Natural Resources in China. The boundaries of the base map have not been modified.

ESV data for the years 2011, 2019, and 2022 were selected to analyze the spatial pattern within the PRD urban agglomeration (Figure 6). Overall, while there are significant differences in ESV among the cities (42), the spatial pattern remained relatively stable, exhibiting a distribution of “low in the east and high in the west, high in the north and low in the south.” A more detailed examination of the three selected years reveals that cities such as Zhaoqing, Huizhou, and Jiangmen consistently maintained relatively high ESV, suggesting these cities have performed well in maintaining and enhancing ESV. This may be attributed to their larger endowments of natural protected areas, extensive forest coverage, and abundant water resources, which provide a solid foundation for ecosystem services. In contrast, more economically advanced cities with higher degrees of industrialization and urbanization, such as Guangzhou, Shenzhen, and Dongguan, exhibited relatively lower ESV. This is likely associated with factors such as intensive land-use change, urban expansion, and environmental pollution, which have exerted negative impacts on their ecosystem services.

### 4.3 Spatial and temporal evolution of the coupling coordination degree between high-quality tourism development and ESV in the PRD urban agglomeration

#### 4.3.1 Temporal evolution of coupling coordination degree in the PRD urban agglomeration

**Figure 7:**
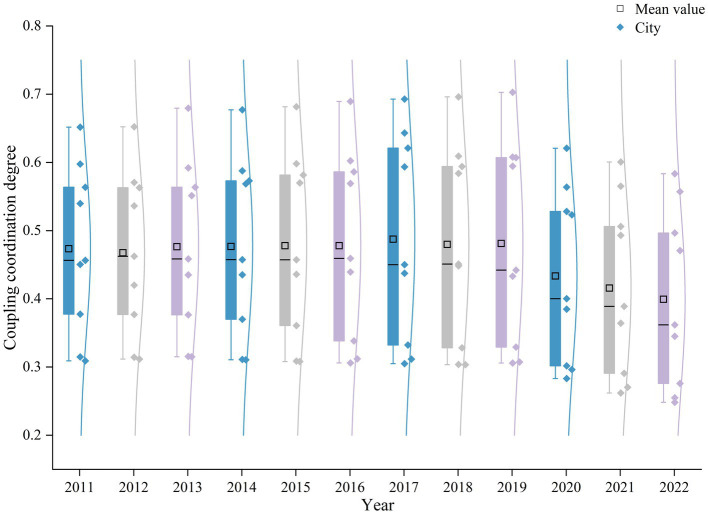
Overall trend of CCD between High-quality tourism development and ESV in the PRD urban agglomeration from 2011 to 2022.

A further analysis was conducted on the CCD between high-quality tourism development and ESV in the PRD urban agglomeration from 2011 to 2022. Figure 7 presents a box plot of the CCD.

As shown in the Figure 7, the mean value of the CCD primarily fluctuated between 0.39 and 0.49 during the study period, exhibiting an overall trend of a slow increase followed by a decline. According to the classification table for CCD (Table 3), the average level for the PRD urban agglomeration from 2011 to 2021 was categorized as being “borderline disorder.” However, due to the severe impact of the COVID-19 pandemic, the level dropped to “mild disorder” in 2022. This indicates that, except for the disorder observed in 2022, the average CCD in the PRD urban agglomeration has consistently been in an “run-in” stage. And the CCD among cities within the PRD urban agglomeration varies significantly.

**Figure 8:**
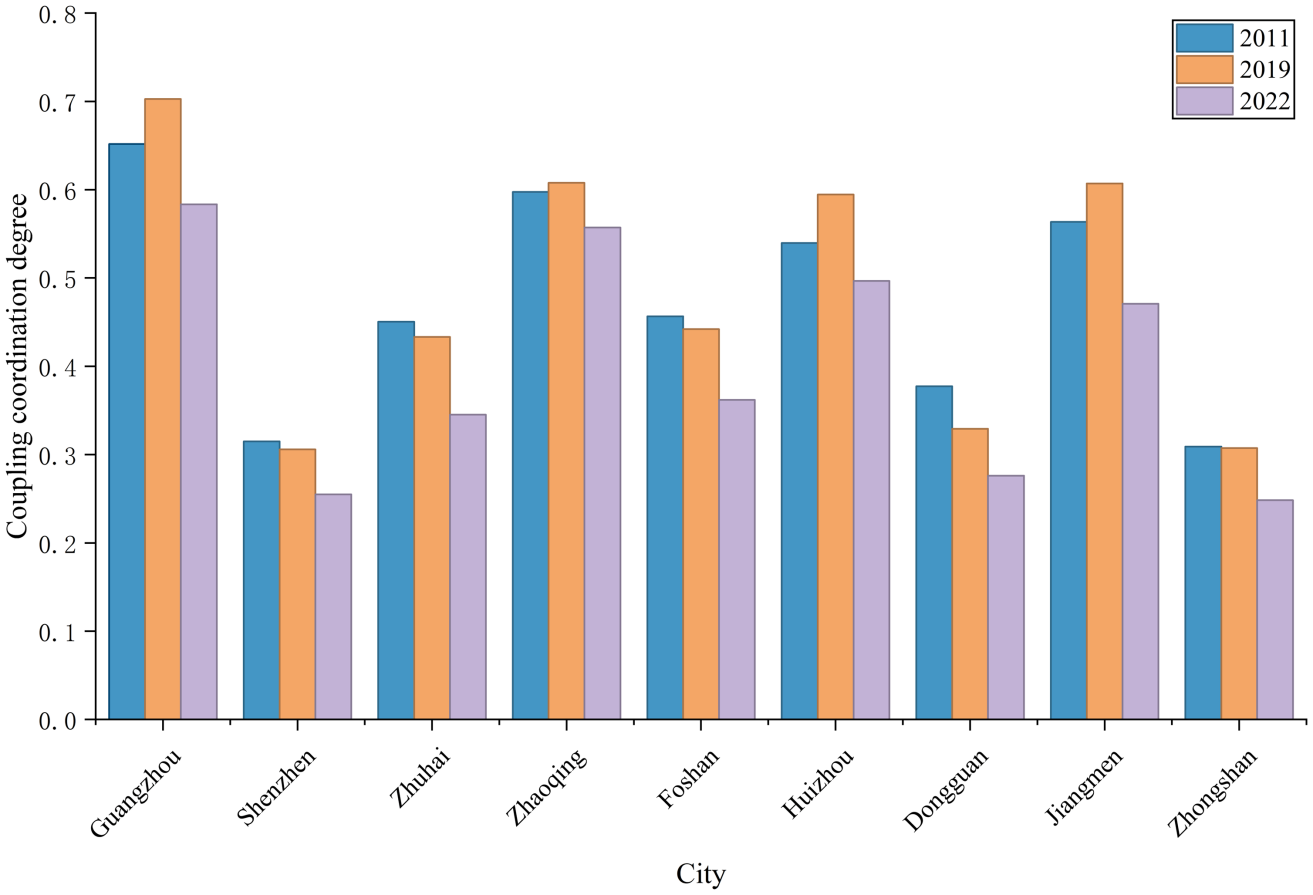
Overall trend of CCD between high-quality tourism development and ESV in the PRD urban agglomeration at the city level from 2011 to 2022.

At the city level, two distinct trend categories for the CCD emerged across the PRD urban agglomeration during the study period. The first category, which includes Guangzhou, Zhaoqing, Huizhou, and Jiangmen, exhibited a trend of an initial increase followed by a decrease in their CCD. The second category, comprising Shenzhen, Zhuhai, Foshan, Dongguan, and Zhongshan, displayed a fluctuating downward trend. Furthermore, the disparity in the CCD among the cities first widened and then narrowed. The difference between the maximum and minimum values was 0.34 in 2011, increased to 0.40 in 2019, and then decreased back to 0.34 in 2022. This narrowing of the gap is likely attributable to the impact of the COVID-19 pandemic, as tourism development in all cities was affected to some extent, leading to a reduction in the overall disparity (Figure 8).

#### 4.3.2 Spatial distribution of coupling coordination degree in the PRD urban agglomeration

**Figure 9:**
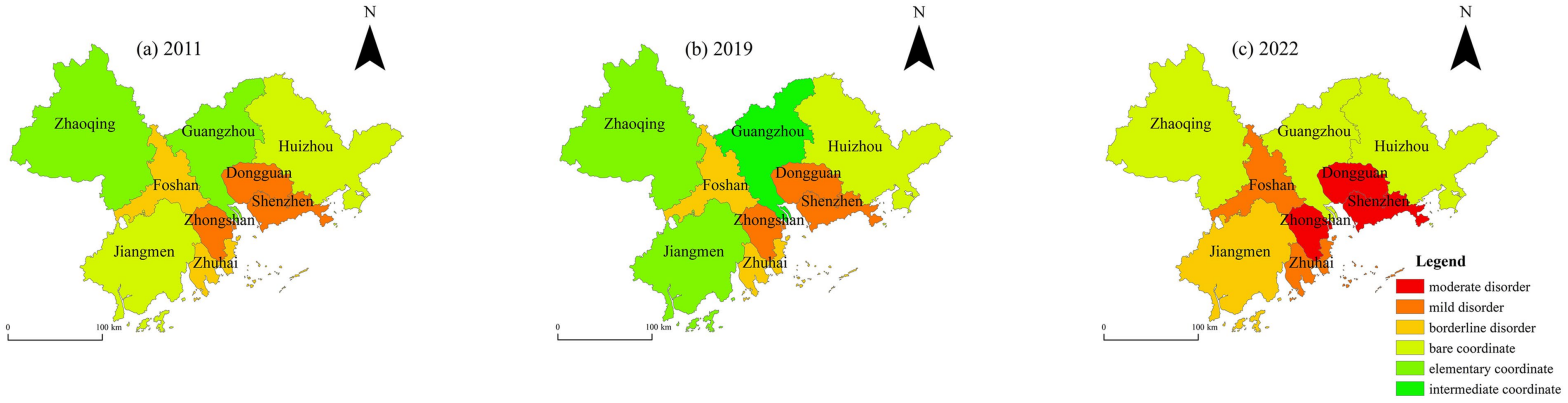
Temporal evolution of CCD between high-quality tourism development and ESV in the PRD urban agglomeration from 2011 to 2022. This map is based on the standard map with the review number GS (2019)1822 downloaded from the Standard Map Service Website of the Ministry of Natural Resources in China. The boundaries of the base map have not been modified.

A spatial analysis of the CCD between high-quality tourism development and ESV in the PRD for the years 2011, 2019, and 2022 was conducted, as illustrated in Figure 9. The spatial distribution of the CCD in the PRD urban agglomeration exhibits a pattern of being “high in the north and low in the south, high in the west and low in the east.” From 2011 to 2019, the overall spatial pattern of the CCD remained relatively stable, with most cities in the “run-in” stage. From 2019 to 2022, a majority of cities transitioned into a disorder stage.

Specifically, in 2011, the overall CCD in the PRD urban agglomeration was relatively low, with most cities in the states of “bare coordinate” and “borderline disorder.” In 2019, the CCD of the PRD urban agglomeration had improved compared to 2011. Guangzhou began to transition from “elementary coordination” toward “intermediate coordination,” and Jiangmen advanced from “barely coordinated” to “elementary coordination,” indicating an enhanced synergy between high-quality tourism development and ESV. In 2022, due to the impact of the COVID-19 pandemic, the overall CCD declined. Most cities, including Dongguan, Shenzhen, and Zhongshan, were in a state of “moderate disorder.” Guangzhou and Zhaoqing were categorized as “bare coordination,” while Jiangmen regressed from “bare coordination” to “borderline disorder.”

## 5 Analysis of influencing factors

Panel data models can mine information from both cross-sectional and time-series dimensions. Using the specified formula, a random-effects analysis was conducted to examine the influencing factors of the CCD. The results are presented in Table 6.

**Table 6:** Panel model analysis.

Items	RE model
Economic development *(ED)*	0.069**
Urban expansion *(UR)*	−0.279**
Industrial structure *(IS)*	0.128**
Government environmental regulation *(GER)*	0.035*
*R* ^2^	0.719
Intercept	0.534**

* *p* < 0.05, ** *p* < 0.01.

As shown in the table, the overall results indicate that urban expansion has a significant negative correlation with the CCD. Conversely, economic development, industrial structure, and government environmental regulation all demonstrate a significant positive promoting effect. An examination of the R^2^ value reveals that the model has a high goodness-of-fit at 71.9%, indicating strong performance.

The research findings can be interpreted as follows:

(1) Economic Development has a clear positive effect on the CCD (*β*_1_ *= 0.069, p < 0.01*). As the economy grows, it provides more sufficient financial and technological support for the region. This enables greater investment in ecological environmental protection, pollution governance, and the construction of sustainable tourism infrastructure. Consequently, while pursuing economic benefits, the region also gains the capacity to maintain and enhance ecological quality, thereby fostering the synergistic development of high-quality tourism and ecosystem services.
(2) Urban Expansion exhibits a significant negative correlation with the coupling. Coordination degree of high-quality tourism development and ESV (*β*_4_ *= −0.279, p < 0.01*). Urban expansion often entails drastic shifts in land-use patterns, namely the large-scale conversion of natural ecological spaces—such as cropland, wetlands, and forests—into construction land. This directly weakens the provision of ecosystem services, leading to biodiversity loss, diminished soil and water conservation functions, and reduced air purification capacity. This process undermines the high-quality ecological foundation upon which tourism depends, exacerbating the conflict between tourism development and ecological conservation and thus lowering their level of coordination.
(3) Industrial Structure shows the most significant positive effect (*β*_2_ *= 0.128, p < 0.01*). This suggests that as a. regional economy transition from resource-intensive and pollution-heavy secondary industries to a tertiary sector dominated by services, the overall ecological footprint of economic activity is reduced. As a key component of the tertiary sector, high-quality tourism is inherently reliant on a healthy ecological environment. Therefore, the optimization and upgrading of the industrial structure compel the region to focus more on the protection and rational use of ecological resources. This creates a virtuous cycle where economic development and ESV mutually reinforce each other, significantly enhancing the level of coupling coordination.
(4) Government Environmental Regulation also has a positive impact on the coupling. Coordination degree (*β*_3_ *= 0.035, p < 0.05*). By formulating and enforcing strict environmental policies, regulations, and standards, such regulation effectively constrains economic activities that could harm the ecosystem. It guides the tourism industry toward green and low-carbon development and promotes the implementation of ecological restoration and protection projects. This provides a solid institutional guarantee and ecological support for high-quality tourism development, thereby strengthening the coordination between it and ESV.

## 6 Discussion

### 6.1 Comparisons with relevant research results

This study investigates the coupling coordination evolution between high-quality tourism development and ESV, along with its influencing factors. A substantial body of existing research has explored the coupled development of tourism and the ecological environment. On one hand, these studies have typically characterized tourism development using traditional indicator systems such as tourism resilience, tourism economy, or general industry metrics for coupling analysis with the environment. However, as living standards rise, tourism has become a way of healthy living, the public’s demand for tourism has shifted from quantity to quality. Consequently, high-quality tourism development has become a contemporary imperative. On the other hand, in the characterization of the environment, ESV is increasingly being applied in tourism research as a more direct and intuitive measure of ecological quality (43). Despite this progress, there is a relative scarcity of research on this topic at the municipal scale (20). Therefore, the present study contributes to and refines the body of research on the coordinated relationship between tourism development and environmental protection. Of course, it also provides a research perspective for achieving human health and well-being.

In characterizing high-quality tourism development, this study integrates sustainable development theory with the concept of high-quality tourism development, thereby establishing an effective and generalizable indicator system at a theoretical level. For instance, our research employed metrics such as the number of overnight inbound tourists, which enhances the objectivity of measurement in tourism development studies. By calculating the ESV of the PRD region, we found that its ecosystem service value showed a decreasing trend from 2011 to 2022. This is consistent with existing research and requires sufficient attention (44, 45). The findings reveal that the average CCD between high-quality tourism development and ESV in the PRD urban agglomeration is generally in an “run-in phase,” showing a trend of first rising and then falling. This is different from other studies on the coupling between tourism subsystems and ESV. For example, the study of Zhao et al. shows that the CCD of the tourism economy resilience in the PRD gradually improved (20), while in this study, it presented a fluctuating trend. This indicates that high-quality tourism development is not equivalent to pursuing economic benefits but emphasizes the high-level sustainability of tourism development (25). Moreover, our research indicates that the CCD reached its peak in 2019 and then declined. This largely demonstrates that the COVID-19 has impacted the tourism (46), which in turn has affected the degree of coordination.

Regarding the factors influencing the coordinated development of tourism and ESV, many existing studies have employed the obstacle degree model for measurement (10, 20). Those studies have generally shown that economic factors have the greatest impact on the CCD, followed by tourism, social, and natural factors. This study employs a random effects model to conduct a further detailed analysis of the influencing factors, finding that while industrial structure and economic development both significantly affect the CCD, the impact of industrial structure is the most significant. This highlights the critical importance of effective industrial restructuring and leveraging the role of the tertiary sector in promoting the coordinated development of tourism and the environment. Secondly, this study identifies an inhibitory effect of urban expansion on the CCD. According to relevant research (41, 47–49), the PRD urban agglomeration has experienced the fastest rate of urban land expansion and the largest increase in construction land area, while land use types such as cropland, forests, and water bodies have progressively diminished. The reduction in cropland has been particularly pronounced. Predictive studies have indicated that most cities in the region will face “medium-alert” or “high-alert” stages in the future. This trajectory will lead to a future decline in ESV, and it could further negatively affect the CCD, thereby impacting the sustainable development of the PRD urban agglomeration. Therefore, our research provides further evidence corroborating the adverse effect of urban expansion on the coordinated development of the tourism and environmental systems. Thirdly, this study reveals a promoting effect of government environmental regulation on the CCD. We employed a novel method to characterize government environmental regulation (33), thereby expanding the methodological scope in this research area. Furthermore, government environmental regulation promotes regional development on multiple levels. For instance, it has been a key driver in the relocation of polluting industries from the PRD to other regions (50). Thus, our study further underscores the importance of government environmental regulation in the coordinated development of tourism and the environment, indicating the government’s leading role in achieving such coordination.

### 6.2 Policy recommendations

Based on the above findings and discussion, this paper proposes the following policy recommendations for different tourism-related agencies.

#### 6.2.1 For governmental agencies and destination management organizations (DMOs)

Further promote the CCD between regional high-quality. Tourism development and ESV, guiding it toward a coordinated state. Although the level of coupling coordination has improved in recent years, it remains in an “run-in phase.” Moreover, due to the impact of the COVID-19 pandemic, it had fallen into a “disorder stage” by 2022. Therefore, it is essential to intensify efforts to advance high-quality tourism development while actively enhancing the realization of ESV. Through government environmental regulation, scientific green tourism development plans should be formulated, firmly establishing the development philosophy that “lucid waters and lush mountains are invaluable assets.” Furthermore, more policies and measures should be introduced to promote a green tourism economy, encourage businesses to increase investment in green technology research and development, and steer the tourism industry toward a sustainable, green trajectory. This is particularly crucial for cities currently on the verge of dissonance.

Adopt suited, locality-specific strategies to advance a new phase in which high-quality tourism development and ESV in the PRD urban agglomeration mutually reinforce each other. The adjustment, transformation, and upgrading of the industrial structure should be promoted to create a green industry chain for tourism. For cities at different stages of urbanization, targeted strategies must be adopted during the expansion process to mitigate the developmental dissonance it can cause between the ecological environment and the tourism industry. Interconnectivity among cities should be enhanced, and cross-regional cooperation among municipalities and departments should be supported to break down administrative barriers and accelerate the tourism integration process in the PRD urban agglomeration, thereby further improving the CCD. In addition, differentiated strategies should be applied: For cities with high ESV but a lower level of high-quality tourism development, such as Zhaoqing, Jiangmen, and Huizhou, it is necessary to further strengthen the protection of ecological resources to improve ecosystem stability and service functions (51). Following this, ecotourism products should be progressively developed. The protection and development of historical and cultural resources should also be enhanced, integrating them with tourism development. Tourists should be guided to participate in ecological conservation activities to achieve a virtuous interaction between tourism and the environment. For cities with a high level of high-quality tourism development but relatively low ESV, such as Guangzhou, Shenzhen, Zhuhai, and Foshan, as well as for cities with low levels in both aspects, like Dongguan and Zhongshan, it is imperative to further strengthen ecological restoration and rationalize the development and utilization of tourism resources to ensure the recovery of ESV. During the tourism development process, the focus should be on creating ecotourism products and actively encouraging tourist participation in conservation activities.

#### 6.2.2 For tourism enterprises

The positive impact of industrial structure adjustment on the CCD indicates the role of the tertiary industry in promoting the coordinated development of tourism and the environment. As the most representative enterprises in the tertiary industry, tourism enterprises should actively undertake social responsibilities for environmental protection. First, tourism enterprises should demonstrate their initiative, increase investment in green technology innovation, implement energy conservation and emission reduction measures, and promote the construction of green hotels and low-carbon tourist attractions. Moreover, enterprises can develop and provide more eco-tourism products and services that rely on high-quality ecosystems, such as nature study tour, etc. This not only meets the growing demand of people for high-quality tourism experiences but also creates economic value for protecting the ecosystem. In addition, enterprises should strengthen environmental education for tourists, guide tourists to take responsible tourism behaviors, participate in ecological protection activities, and form a virtuous cycle where tourism development promotes environmental protection.

### 6.3 Limitations and suggestions for future research

Of course, this study is not without its limitations. The primary limitation of this study lies in the unavailability of complete data beyond 2022, which constrains our analysis in two critical ways. First, the inability to measure the CCD for 2023 and the subsequent post-pandemic era means the current findings cannot capture the latest CCD level between high-quality tourism development and the ESV, potentially affecting the long-term validity of the observed trends. Second, the lack of effective variables to characterize the impact of the COVID-19 pandemic limits our understanding of the key disruptive factor influencing the coordination during the study period. Therefore, future research should prioritize integrating post-2023 data to validate and extend the temporal scope of this study, while also employing cross-scale comparisons and mixed-method approaches to deepen the causal inference and generalizability of the findings.

## 7 Conclusion

Focusing on urban agglomerations from the perspective of sustainable development theory and incorporating China’s five concepts for development, this study constructed an evaluation indicator system for high-quality tourism development. It conducted a comprehensive measurement of the CCD between high-quality tourism development and ESV in the PRD urban agglomerations from 2011 to 2022, and analyzed its spatial–temporal evolution patterns and influencing factors. The conclusions are as follows:

First, from an overall perspective, the level of high-quality tourism development in the. PRD urban agglomerations exhibited a trend of an initial increase followed by a decrease between 2011 and 2022. The ESV showed a continuous decline. The decrease in both high-quality tourism development and ESV was particularly significant during the 2020–2022 period. The CCD between the two systems also followed a trend of first rising and then falling.
From a temporal perspective, the average CCD within the region experienced a steady, slight increase from 2011 to 2019, followed by a gradual decline from 2020 to 2022. Except for 2022, which was in a “disorder stage,” the average coupling degree in the PRD urban agglomerations remained in an “run-in stage” from 2011 to 2021. The CCD trends for individual cities were similar, showing an increase followed by a decrease, while the CCD gap among the cities continuously narrowed. From a spatial perspective, the distribution of the CCD was characterized as “high in the north and low in the south, high in the west and low in the east.” Over the study period, the cities shifted from a general trend toward coordination to a trend toward disorder. Notably, the CCD of all cities improved in 2019, but subsequently, due to the impact of the pandemic, they gradually moved toward a state of disorder.
Regarding the influencing factors, this study utilized four indicators—economic development, industrial structure, government environmental regulation, and urban expansion—for measurement. The panel model analysis revealed that, except for urban expansion, which had an inhibitory effect on the development of the CCD, the other three factors all played a significant promoting role.

## Data Availability

The original contributions presented in the study are included in the article/supplementary material, further inquiries can be directed to the corresponding author.
