# Correction: From pursuing quantity to pursuing quality: exploring the coupling coordination and influencing factors of high-quality tourism development and ecosystem service value in the Pearl River Delta urban agglomeration of China

**DOI:** 10.3389/fpubh.2026.1769307

**Published:** 2026-01-22

**Authors:** Hao Yao, Zeduo Zou, Yiting Zhu, Chunshan Zhou

**Affiliations:** 1 College of Tourism, Xinjiang University, Urumqi, China; 2 School of Geography and Planning, Sun Yat-Sen University, Guangzhou, China

**Keywords:** high-quality tourism development, ecosystem services value, coupling coordination, PRD urban agglomeration, sustainable development theory

There was a mistake in Table 1 as published. In the submitted paper all the references in the “source” and supplement the unit for “Indicator Explanation” sections were included. However, the published article contained many blank spaces. The corrected Table 1 appears below.

**Table 1:** Construction of the index system for high-quality tourism development based on the sustainable development theory.

**Dimension 1**	**Dimension 2**	**Indicator**	**Indicator explanation**	**Indicator attribute**	**Source**
Economical level	Tourism innovation development	Tourism investment and research expenditure	R&D expenditure × (total tourism revenue/GDP) (10,000 yuan)	+	(36)
		Tourism innovation products	The number of tourism patents held by 10,000 people (pieces)	+	(36)
	Tourism openness level	The number of inbound arrivals	The number of overnight inbound visitors (10,000 people)	+	(22)
		Inbound consumption	Inbound tourism revenue (100 million yuan)	+	(8)
	Tourism area coordination	Contribution of tourism to the economy	Domestic tourism revenue (100 million yuan)	+	(9)
			The proportion of tourism revenue in the GDP (%)	+	(9)
		Contribution of tourism to employment	The proportion of tourism industry workers in the total number of employees (%)	+	(37)
		The contribution of tourism industry linkage	The proportion of tourism revenue in the output value of the tertiary industry (%)	+	(8)
Social cultural level	Tourism products and services sharing	Density of high-quality tourism resources	Number of 4A and 5A-level scenic spots (places)	+	(37)
		High-quality accommodation supply for tourism	Number of four-star and five-star hotels (places)	+	(37)
		Tourism public transportation services	The number of taxis in operation per capita at the end of the year (in units of vehicles)	+	(36)
			The number of actual public transport vehicles in operation per capita at the end of the year (in units of vehicles)	+	(36)
		Tourism Public Health Services	The number of public toilets (facilities) per 10,000 people (places)	+	(32)
		Tourism cultural facility services	Number of museums (places)	+	(22)
			Number of public libraries (places)	+	(22)
			Number of art performance groups and institutions (places)	+	(22)
Environmental level	Tourism green development	Park green space area	Per capita park green space area (m^2^)	+	(32)
		Greening of the built-up area	Green coverage rate of built-up area (%)	+	(32)
		Level of air pollution	Annual average PM2.5 concentration (μg/m^3^)	-	(36)
		Garbage harmless treatment	Garbage harmless treatment (%)	+	(32)
		Wastewater treatment	Wastewater treatment rate (%)	+	(36)

The original version of this article has been updated.

